# Persistent Anticoagulation and Severe Upper Gastrointestinal Bleeding in a Patient With Factor V Leiden: A Case of the Warfarin Reservoir Phenomenon

**DOI:** 10.7759/cureus.91638

**Published:** 2025-09-04

**Authors:** Yasir Kammawal

**Affiliations:** 1 Internal Medicine, Isle of Wight (IOW) NHS Trust, Isle of Wight, GBR

**Keywords:** factor v leiden, gastrointestinal bleeding, pharmacokinetics, thrombophilia, warfarin

## Abstract

We report a case of a 58-year-old man with atrial fibrillation who presented with hematemesis and melena despite reporting that he had discontinued warfarin therapy three months earlier. On admission, he was hypotensive and tachycardic, with laboratory tests showing hemoglobin of 6.8 g/dL and INR of 4.2. A plasma assay confirmed detectable warfarin levels, suggesting a persistent anticoagulant effect despite discontinuation. Endoscopy revealed a bleeding gastric ulcer, which was treated successfully with endoscopic hemostasis. The patient required a blood transfusion, vitamin K, and fresh frozen plasma for INR reversal. Further work-up revealed a heterozygous factor V Leiden mutation, creating a paradoxical clinical situation of both thrombotic predisposition and major hemorrhage. This case highlights the importance of recognizing unusual pharmacokinetic persistence of warfarin, the so-called “warfarin reservoir” phenomenon, and the clinical challenge posed by coexisting inherited thrombophilia. Awareness of this scenario can aid in timely diagnosis, appropriate reversal strategies, and multidisciplinary decision-making for long-term anticoagulation management.

## Introduction

Anticoagulant-related gastrointestinal (GI) bleeding is a frequent and potentially life-threatening complication of warfarin therapy, with an annual incidence ranging from 1% to 4% in anticoagulated patients [1,2]. These figures are largely derived from large registry-based and community cohorts, where elderly patients and those with multiple comorbidities demonstrated the highest risk [3]. Warfarin is a vitamin K antagonist that inhibits hepatic synthesis of clotting factors II, VII, IX, and X. Its anticoagulant effect is monitored using the international normalized ratio (INR), with therapeutic ranges typically between 2.0 and 3.0. Because warfarin has a half-life of approximately 36-42 hours, anticoagulant activity usually resolves within three to five days after discontinuation. Persistence of anticoagulation beyond this period is unusual and prompted consideration of alternative mechanisms.

One proposed explanation is the “warfarin reservoir phenomenon,” in which warfarin accumulates in tissues such as adipose and continues to exert anticoagulant effects despite cessation [4,5]. Although direct human case series are limited, pharmacokinetic and animal distribution studies support the potential for depot-like tissue sequestration [6]. This makes the reservoir hypothesis an exceptional consideration when conventional explanations for prolonged anticoagulation are excluded.

Factor V Leiden (FVL) mutation, the most common inherited thrombophilia, is present in approximately 3-8% of Caucasian populations [7,8]. More recent epidemiologic reviews confirm that heterozygosity is particularly prevalent in individuals of European ancestry [9]. FVL predisposes to venous thromboembolism but does not independently cause bleeding. The coexistence of FVL in a patient requiring anticoagulation who then develops life-threatening GI bleeding due to reservoir warfarin represents a rare and paradoxical clinical scenario. This case highlights the importance of considering both unusual pharmacokinetic effects and inherited thrombophilia when managing anticoagulation-related complications.

## Case presentation

A 58-year-old male with a background of atrial fibrillation and previously prescribed warfarin presented to the emergency department with sudden-onset hematemesis and melena. He reported that he had discontinued warfarin approximately three months prior to admission. Three days before presentation, he developed dark stools, which progressed to frank melena, followed by a large episode of hematemesis on the day of admission. His past medical history included hypertension and a prior ischemic stroke. There was no history of alcohol misuse or chronic liver disease. The patient denied unintentional weight loss or other constitutional symptoms in the months prior to presentation.

On examination, the patient was pale, hypotensive (blood pressure 92/58 mmHg), and tachycardic (118 beats per minute). Abdominal examination was unremarkable, with no signs of chronic liver disease. Digital rectal examination confirmed melena.

Initial laboratory results are summarized in Table 1. Hemoglobin was 6.8 g/dL, hematocrit 21%, platelets 185 ×10⁹/L, INR 4.2, urea 15 mmol/L, and creatinine 106 μmol/L. A plasma warfarin assay confirmed detectable circulating drug levels despite the patient’s claim of discontinuation. Other potential contributors-including covert warfarin ingestion, drug interactions, and liver dysfunction-were reviewed and excluded based on history, medication reconciliation, and laboratory testing.

**Table 1:** Patient’s admission laboratory results with corresponding normal reference ranges.

Tests	Results	Reference ranges
Hemoglobin	6.8 g/dL	13.5-17.5 g/dL
Hematocrit	21%	40-50%
Platelets	185 ×10⁹/L	150-400 ×10⁹/L
INR	4.2	0.9-1.2
Urea	15 mmol/L	2.5-7.5 mmol/L
Creatinine	106 µmol/L	60-110 µmol/L

The patient was resuscitated with intravenous fluids and transfused with packed red blood cells. He received intravenous vitamin K and fresh frozen plasma for INR reversal. Urgent upper gastrointestinal endoscopy revealed an actively bleeding gastric ulcer, which was successfully treated with endoscopic hemostasis using adrenaline injection and hemoclip application.

**Figure 1:**
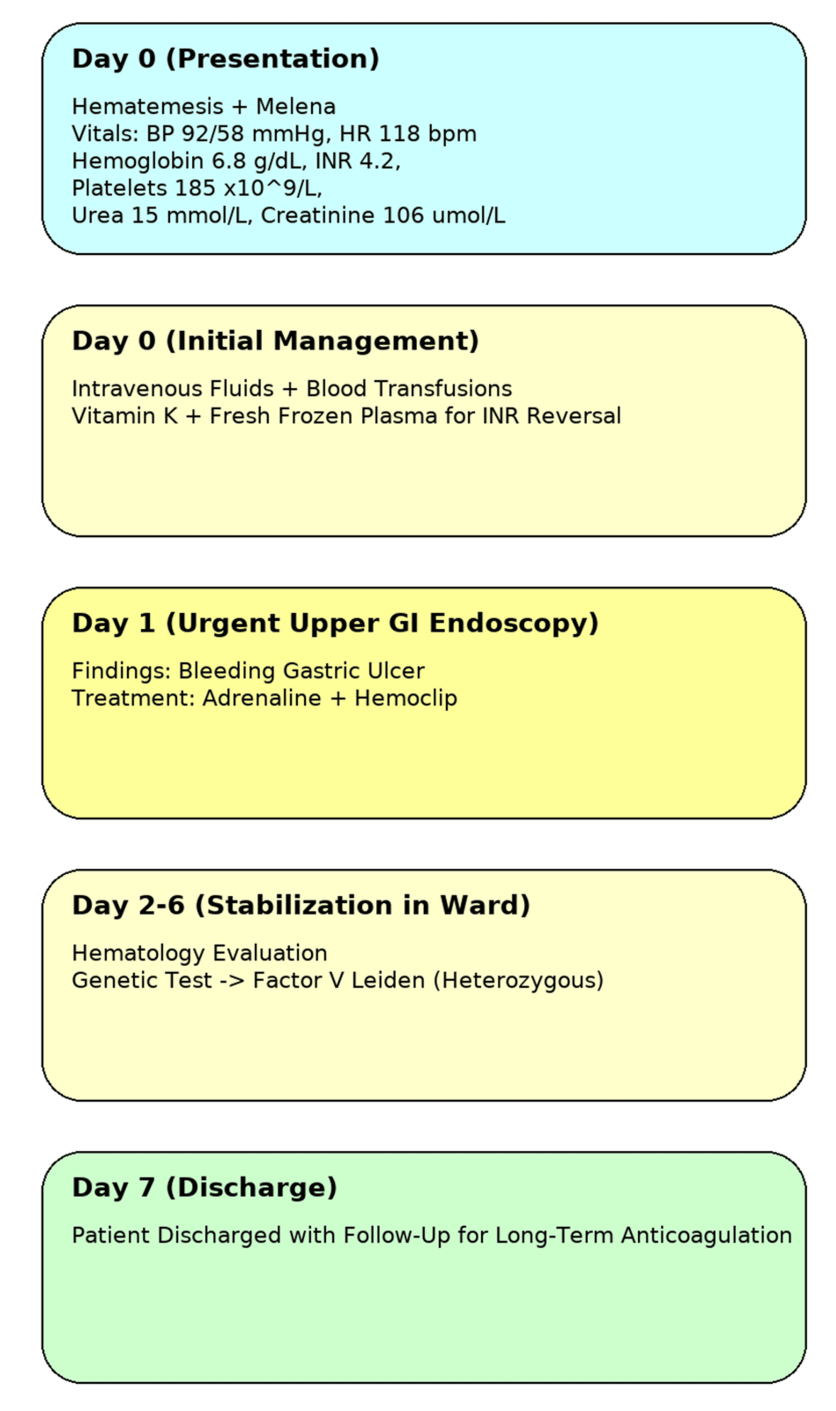
Patient’s clinical course from presentation and laboratory findings to treatment, genetic testing, and discharge.

Following stabilization, the patient underwent thrombophilia screening due to his complex anticoagulation history, which revealed a heterozygous factor V Leiden mutation. He was discharged after seven days of inpatient care, with outpatient hematology follow-up arranged to reassess long-term anticoagulation strategy. At two-week and one-month follow-up, the patient remained clinically stable with hemoglobin recovery and normalization of INR. Ongoing management focused on individualized anticoagulation risk-benefit reassessment given his thrombophilic background. An overview of the clinical course is illustrated in Figure 1.

## Discussion

Anticoagulation-associated gastrointestinal (GI) bleeding remains a major cause of hospital admissions, with warfarin being one of the most frequently implicated agents. The annual risk of major bleeding in patients on warfarin ranges from 1% to 4%, and GI bleeding represents nearly half of these events [1,2,7]. Most bleeding episodes are explained by supratherapeutic INR, poor monitoring, or drug-diet interactions. In our patient, however, anticoagulation persisted despite reported discontinuation of warfarin three months earlier, suggesting an atypical pharmacokinetic mechanism consistent with the “warfarin reservoir” hypothesis [4,8].

The warfarin reservoir phenomenon refers to the deposition of warfarin into tissues, such as adipose tissue, with subsequent slow release back into the circulation. While described in case reports, this may explain prolonged anticoagulant activity in some patients even after therapy has been discontinued [4,8,9]. Typically, INR returns to normal within three to five days of stopping warfarin, yet in this case, the INR remained markedly elevated (4.2) months after discontinuation, highlighting the unusual nature of this presentation. This persistence directly influenced management, as vitamin K and fresh frozen plasma were administered to reverse anticoagulation, while the combination of severe anemia and ongoing hemodynamic compromise guided transfusion requirements and prompted urgent endoscopic intervention.

The coexistence of a heterozygous factor V Leiden (FVL) mutation in this patient added to the complexity of management. Factor V Leiden is the most common inherited thrombophilia, present in approximately 3-8% of Caucasians [5,6,9]. It is a prothrombotic condition rather than a bleeding disorder. In this case, the paradox arose when a genetic predisposition to thrombosis coincided with severe hemorrhage due to persistent warfarin effect.

Management of such patients requires a careful balance between preventing thromboembolic events and minimizing hemorrhagic complications. In our case, the immediate priorities included stabilization with blood transfusion, INR reversal, and endoscopic control of bleeding. After discharge, the patient remained stable, with recovery of hemoglobin and normalization of INR at follow-up, allowing for individualized reassessment of anticoagulation needs in view of his thrombophilic background. Long-term management necessitates careful risk-benefit evaluation and multidisciplinary input [2,3,7].

This case highlights the following two important clinical lessons: first, that persistent anticoagulation despite discontinuation of warfarin can occur and should be considered when INR remains elevated without recent dosing; and second, that inherited thrombophilia, such as factor V Leiden, may complicate anticoagulation decisions and mandate multidisciplinary involvement in patient care. A limitation of this report is the absence of formal weight documentation, although the patient did not report unintentional weight loss, and no clinical features of cachexia were observed. As weight may play a role in the context of adipose tissue distribution and the reservoir hypothesis, this omission is acknowledged for completeness.

## Conclusions

This case illustrates the rare but clinically significant persistence of warfarin anticoagulation despite reported discontinuation, consistent with the proposed “warfarin reservoir” effect. As this mechanism remains hypothetical and has only been described in a few reports, it should be considered exceptional and only after common explanations have been excluded. The coexistence of the factor V Leiden mutation created a paradoxical scenario of heightened thrombotic risk alongside life-threatening bleeding. Clinicians should remain vigilant when INR values are unexpectedly elevated, even in patients denying recent anticoagulant use. A multidisciplinary approach involving gastroenterology, hematology, and internal medicine is essential to ensure safe management and guide individualized long-term anticoagulation strategies.
